# Supplementary material for: Pot1 promotes telomere DNA replication via the Stn1-Ten1 complex in fission yeast
Source: Nucleic Acids Res. 2023 Nov 11;51(22):12325–36. doi: 10.1093/nar/gkad1036 (PMC10711446; doi:10.1093/nar/gkad1036)
Supplement: gkad1036_Supplemental_Files [file gkad1036_supplemental_files.zip › Borges_SupMaterial_NAR_R1V3.pdf]

Figure S1

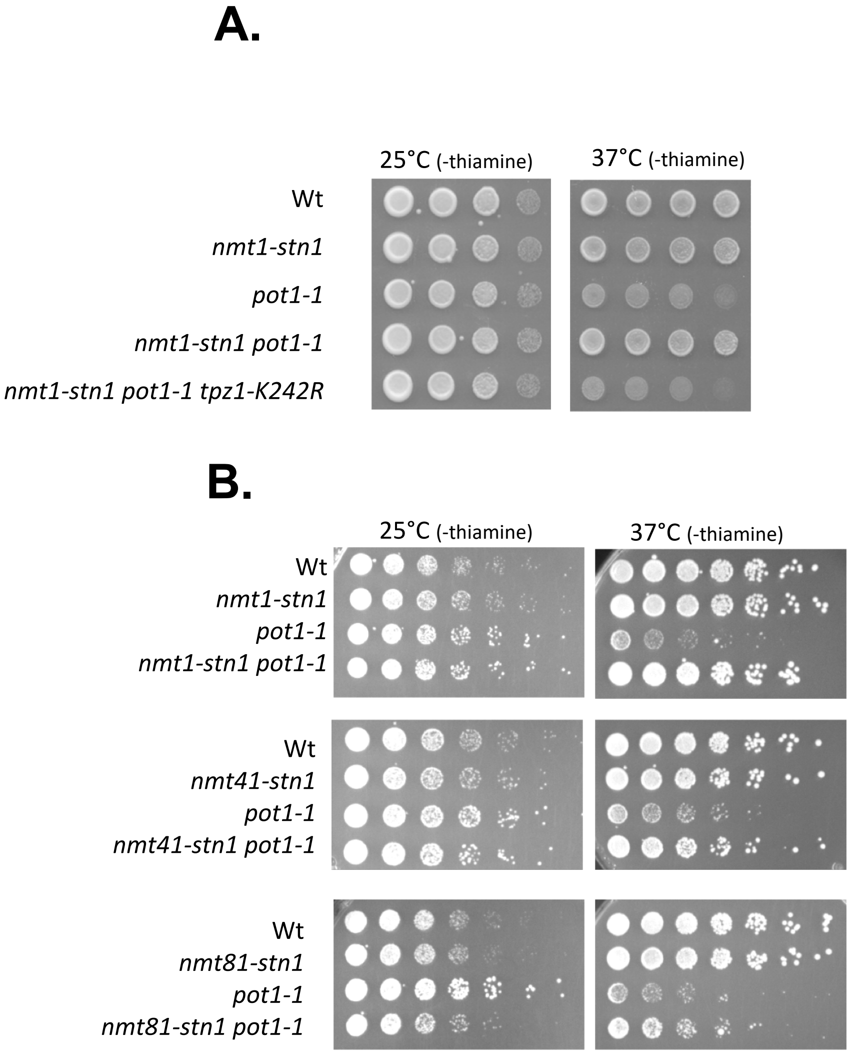

Figure S1. Overexpression of Stn1 rescues *pot1-1* cells viability.
A.) The mutation Tpz1-K242R that prevents the sumoylation of Tpz1 and the recruitment of Stn1 at telomeres abolishes the growth rescue of *pot1-1* cells by overexpression of Stn1 at 37°C. **B.)** Different levels of Stn1 overexpression in *pot1-1* cells were tested and all of them rescued *pot1-1* cells viability. Spot Assay was performed in PMG solid media which lacks thiamine and incubated at 25°C and 37°C.

Figure S2

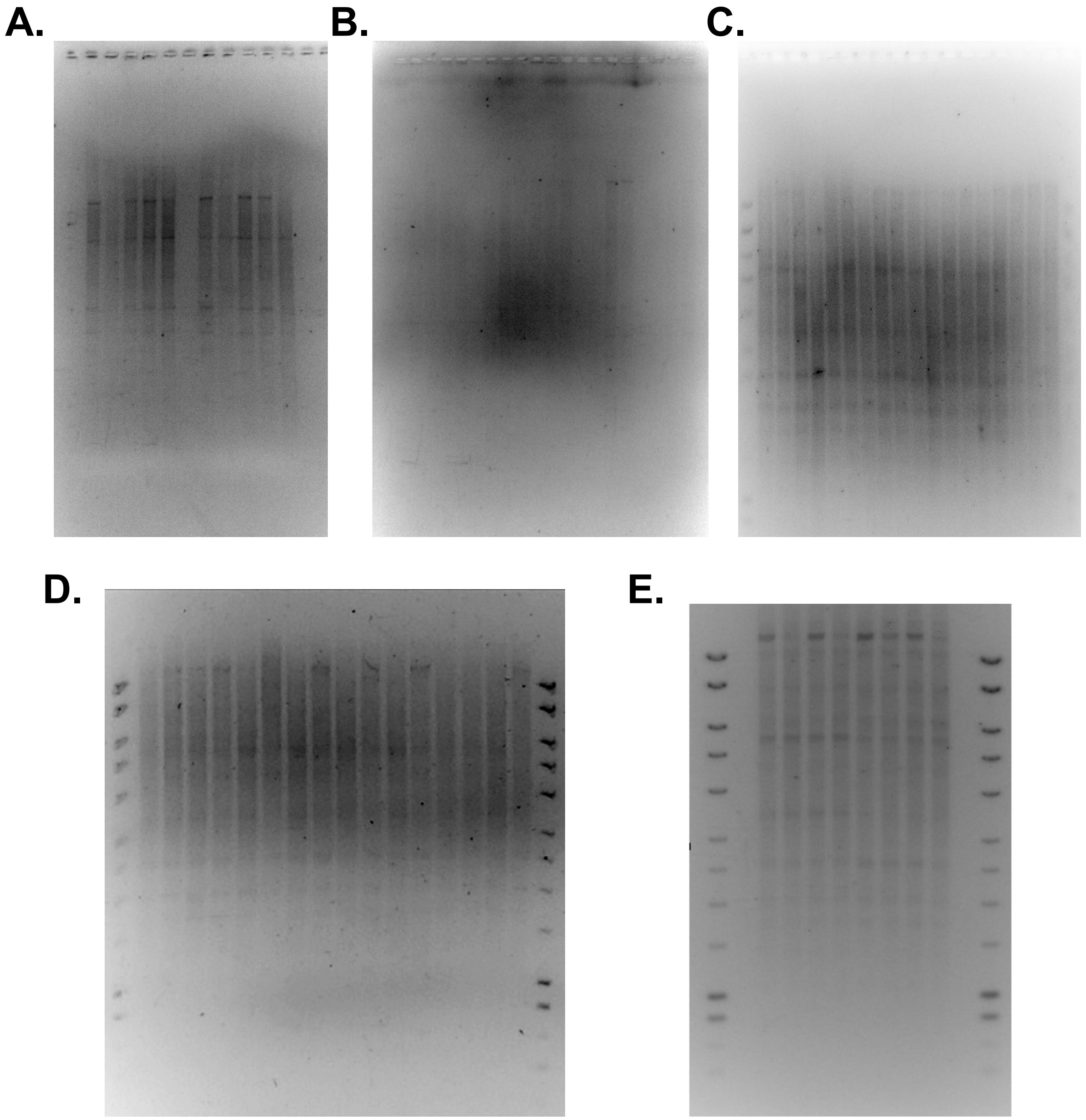

Figure S2. Equal loading of telomere Southern blots evaluated by whole genome EcoRI digestion. All gels were pre-run, quantified using REDSAFE signal and equally loaded before transfer onto membranes. A.) Genomic DNA of Figure 1C. B.) Genomic DNA of Figure 2C. C.) Genomic DNA of Figure 2F. D.) Genomic DNA of Figure 4C. E.) Genomic DNA of Figure 5B.

Figure S3

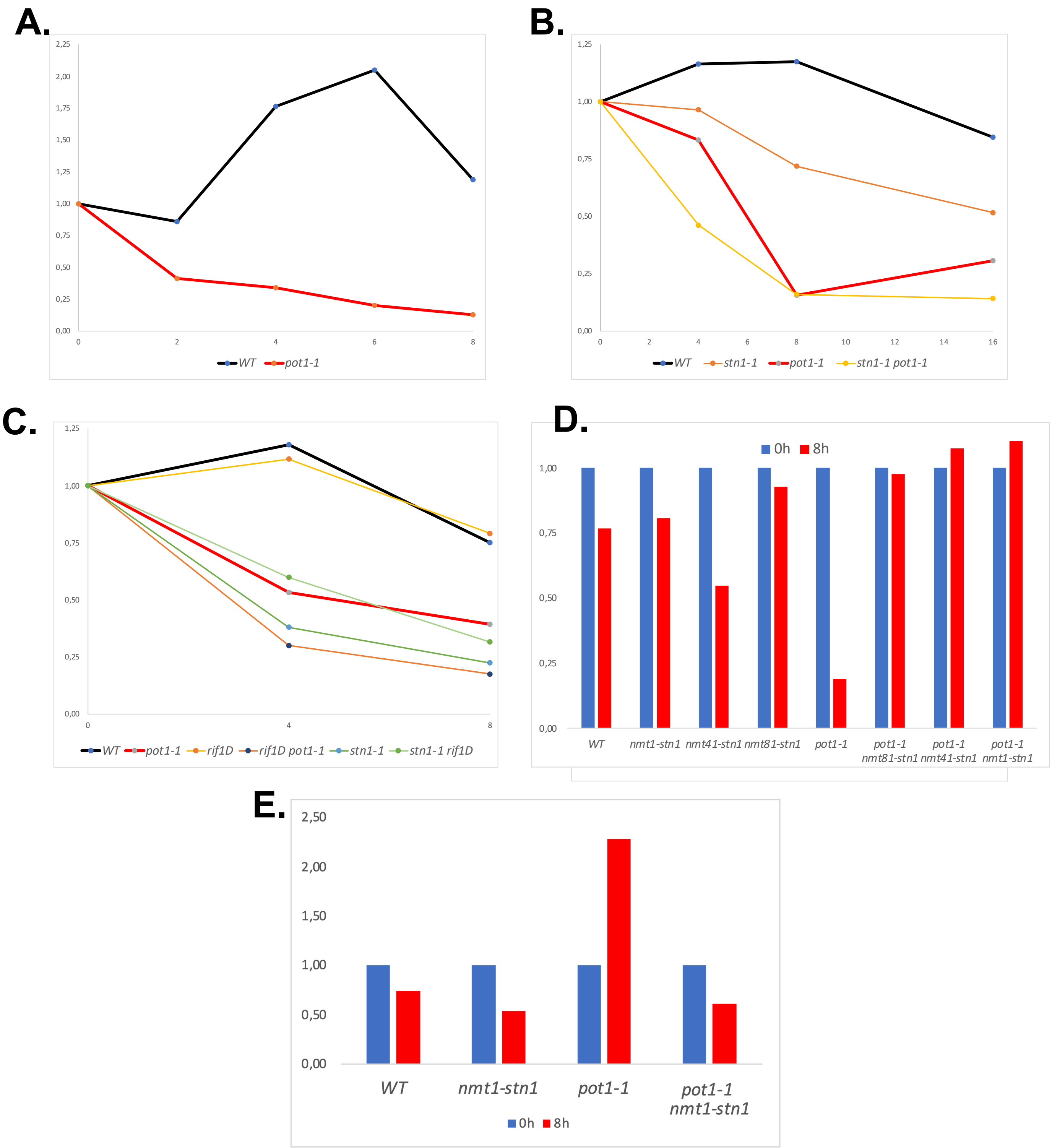

Figure S3. Quantifications of telomere signal. Southern blots were quantified using ImageJ and telomere signal was normalized against loading controls. A.) Southern blot of Figure 1C. B.) Southern blot of Figure 2C. C.) Southern blot of Figure 2F. D.) Southern blot of Figure 4C. E.) In gel hybridization of Figure 4D.
