# Supplementary material for: Pot1 promotes telomere DNA replication via the Stn1-Ten1 complex in fission yeast
Source: Nucleic Acids Res. 2023 Nov 11;51(22):12325–36. doi: 10.1093/nar/gkad1036 (PMC10711446; doi:10.1093/nar/gkad1036)
Supplement: gkad1036_Supplemental_Files [file gkad1036_supplemental_files.zip › Table S1_R1 .pdf]

Supplemental materials

Table S1: Fission yeast strains used in this study

Name	Strain	Genotype	Source
Wt	MGF10	*h- ade6-M210 his3-D1 leu1-32 ura4-D18*	R. McIntosh Lab
pot1-1	MGF1449	*h- ade6-M210 his3-D1 leu1-32 ura4-D18 pot1-1-GFP:KanMX6*	J. Cooper Lab
pot1-GFP	SC922	*H+ ade6-M210 leu1-32 ura4-D18 pot1-GFP:KanMX6*	K. Tomita Lab
exo1Δ	MGF293	*h- ura4-D18 exo1::ura4*	O. Fleck Lab
pot1-1 exo1Δ	MGF2914	*h-ade6-M210 his3-D1 leu1-32 ura4-D18 pot1-1-GFP:kanMX6 exo1::hphMX*	This Study
nmt1-3x-stn1	MGF2377	*h- ade6-M210 his3-D1 leu1-32 ura4-D18 kanMX6:nmt1-3X-stn1*	MGF10
nmt1-41x-stn1	MGF2378	*h- ade6-M210 his3-D1 leu1-32 ura4-D18 kanMX6:nmt1-41X-stn1*	MGF10
nmt1-81x-stn1	MGF2379	*h- ade6-M210 his3-D1 leu1-32 ura4-D18 kanMX6:nmt1-81X-stn1*	MGF10
nmt1-3x-stn1 pot1-1	MGF2949	*h? ade6-M210 his3-D1 leu1-32 ura4-D18 kanMX6:nmt1-3X-stn1 pot1-1-GFP:kanMX6*	This Study
nmt1-41x-stn1 pot1-1	MGF2950	*h? ade6-M210 his3-D1 leu1-32 ura4-D18 kanMX6:nmt1-41X-stn1 pot1-1-GFP:kanMX6*	This study
nmt1-81x-stn1 pot1-1	MGF2952	*h? ade6-M210 his3-D1 leu1-32 ura4-D18 kanMX6:nmt1-81X-stn1 pot1-1-GFP:kanMX6*	This study
nmt1-3x-stn1 pot1-1 tpz1-K242R	SC1552	*h? ade6-M210 his3-D1 leu1-32 ura4-D18 kanMX6:nmt1-3X-stn1 pot1-1-GFP:kanMX6 tpz1-K242R:hphMX6*	This study
stn1-1	MGF2707	*h- his3-D1 leu1-32 ade6-M210 ura4-D18 stn1-1*	F.Ishikawa Lab
pot1-1 stn1-1	*MGF2931*	*h+ pot1-1-GFP::KanMX6 stn1-1*	*This study*
pot1-1 rif1Δ	*MGF2924*	*h+ pot1-1-GFP::KanMX6 rif::hph*	This study
stn1-1 rif1Δ	MGF2954	*h- ade6-M21? his3-D1? leu1-32 ura4-D18 stn1-1 rif1::hphMX6*	This study
tpz1-myc pot1-GFP	SC1525	*h- leu1-32 ura4-D18 tpz1-myc :kanMX6 pot1-GFP:KanMX6*	This study
tpz1-myc pot1-1	SC1526	*h+ leu1-32 ura4-D18 tpz1-myc :kanMX6 pot1-1-GFP:KanMX6*	This study
Wt + pREP41-empty	MGF2665	*h? ade6-M210 his3-D1 leu1-32 ura4-D18 pREP41-nmt41-empty-Leu2*	MGF10
Wt + pREP41-pol1	MGF2666	*h? ade6-M210 his3-D1 leu1-32 ura4-D18 pREP41-nmt41-pol1-Leu2*	MGF10
pot1-1 + pREP41-empty		*h+ ade6-M210 his3-D1 leu1-32 ura4-D18 pot1-1-GFP:kanMX6 pREP41-nmt41-empty-Leu2*	This Study
pot1-1 + pREP41-pol1		*h+ ade6-M210 his3-D1 leu1-32 ura4-D18 pot1-1-GFP:kanMX6 pREP41-nmt41-pol1-Leu2*	This Study
